# Mr. Carmichael's Reply to Mr. Guthrie

**Published:** 1819-04-01

**Authors:** 


### *Mr. CARMICHAEL's REPLY to Mr. GUTHRIE.*

I cannot enter into an examination of Mr. Guthrie's remarks on my late work upon Venereal Diseases, without first testifying my sense of the candid, polite, and creditable manner in which he has discussed the subject, the gratification I feel, that the particulars are so few in which we differ from each other, and my regret that there is a point, however unimportant, in which we are not agreed. But it is satisfactory to find that his objections do not lie against the practice I recommend, but almost exclusively against the doctrine of the plurality of venereal poisons, which I have been led by the strongest reasons to adopt.

But convinced as I am, by a vast multiplicity of evidence, of the truth of this opinion, yet so little do I value it, in comparison of the practical results to which it led me, that throughout my work, I adverted but little to this part of my subject, and confined myself altogether to points of practice, except so far as it was necessary to the explanation of my mode of discriminating the dangerous and malignant species of venereal diseases from the more mild and manageable. Indeed, my most strenuous endeavours, and I thought they could scarcely be mistaken, were directed to convince surgeons of the necessity of paying more attention than was customary, to the distinctive characters and appearances of both primary and constitutional symptoms in venereal complaints.

I had satisfied myself that our diagnosis and mode of treatment ought to be regulated in a great measure by those very characters and appearances, no matter whether we consider them as arising from one poison, or from many, or whether they are owing to peculiarities of constitution, or to those causes assigned by Mr. Guthrie, in his " Remarks," when he says, " I am, then, for the present, disposed to refrain from believing in a plurality of morbid poisons, each invariably producing a disease of one distinct character, or of one specific poison modified by the powers of the constitution, or of the part; *but rather to place reliance in the combined opinion of different morbid poisons (to use the common term) being generated, in different people, and stages and states of disease, under circumstances which I no more understand, than others do of the nature of the syphilitic poison itself.*"

Whether the views of this enlightened surgeon will serve to remove the obscurity with which this perplexing subject is enveloped, and better explain than any thing hitherto offered, the numerous symptoms and appearances which venereal complaints display, I leave to the judgment of the reader, and shall altogether refrain from making any observations upon them. But, in truth, the question will scarcely be allowed to be decided, until the institution of an enlarged scale of experiments by inoculation; which, as I have observed in my last Essay, " it is not to be expected any man will be so public spirited as to submit to himself, or so unreasonable as to inflict upon others." My reasons for espousing the opinion of a plurality of venereal poisons are given at large in the first chapter of my former essay on the subject, with some additional observations in the first chapter of my late publication, which it would be unpardonable here to repeat.

In the latter Essay, I have detailed a number of cases of the primary phagedenic ulcer, which were all attended (two cases excepted) with a similar eruption; viz. by tubercles, or spots of a pustular tendency, which degenerated into obstinate ulcers. In the two exceptions alluded to, the eruption was papular; and I am inclined to conjecture that the primary ulcers from which it arose, were not originally phagedenic, but became so through irritation or improper management.

From various passages in Mr. Guthrie's remarks, I collect, that he is of opinion that there is no such ulcer as a primary venereal phagedena; and that all ulcers of this description are originally mild, and become afterwards phagedenic, through causes with which we are at present unacquainted. But once they become phagedenic, he believes they affect the constitution in such a way as to produce the train of constitutional symptoms I have described as appertaining to the phagedenic venereal ulcer. I trust this is a faithful though concise account of his opinions, as it would render this paper too long to transcribe the passages from which I collect it.

In support of his views, and in opposition to mine, Mr. Guthrie, in the first place, assures us that he has frequently seen those constitutional symptoms which I had attributed to the phagedenic primary ulcer, succeeding primary ulcers which were originally of a mild nature, and which afterwards became phagedenic; and in the next he argues, that the slowness of the progress of the ulcer in many of the cases which I have detailed, sufficiently evinces that they would not, in their origin, have been phagedenic.

Against Mr. Guthrie's own experience I can have nothing to object, and the point must remain at issue between us, to be determined by future observation; but I cannot admit the accuracy of his views, when he describes the phagedenic ulcer as always necessarily rapid in its progress, for I can aver, (and I am confident that I am not singular in this respect) that I have frequently witnessed the existence of this ulcer during four, six, or eight months, or even longer, creeping slowly from one part to another, alternately healing and ulcerating, and leaving deep furrows, yet without destroying, as it more usually does, the parts it attacks. Anomalous therefore as it appears, we may readily conceive why the opera dancer at Lisbon, mentioned by Dr. Ferguson, should infect many of her paramours with a malignant disease, without being herself under the necessity of discontinuing the public practice of her profession.

"Referring to my first work," Mr. Guthrie observes, "we have a sketch of the progress of the phagedenic ulcer; but we do not find any description of that ulcer from the commencement of ulcerative absorption, as we do of the sloughing ulcer, from the commencement of the sloughing process. Neither is one given in the second book; so that we are left in doubt whether his specific phagedena begins as such, or whether it assumes that character after a certain lapse of time. If the former be the fact, it adds strength to my objections to his cases; if the latter, it is necessary we should be informed of the precise period required for the specific character to show itself."—In answer to this observation, I must acknowledge that it has not been my good fortune to see a phagedenic ulcer at an earlier period than perhaps the second or third week after its commencement, and therefore I cannot aver, from my own experience, whether it presents the precise phagedenic character *ab initio*. But I have happened to witness in one instance, the very commencement of the sloughing ulcer, the particulars of which are detailed in case sixty-eight of my first Essay, and are alluded to by Mr. Guthrie in his Remarks.

The gentleman affected with it, who was an intimate friend, shewed it to me a few minutes after his own attention had been solicited to the part; and at this time it exhibited the appearance of a black mortified speck, not larger than a grain of shot. From this small beginning, it went on, alternately sloughing and ulcerating with a phagedenic edge, until it destroyed the greater part of the prepuce, and a portion of the glans.

After it had existed a few days, it did not differ in appearance from the numerous advanced sloughing ulcers I had seen and treated in the wards of the Dublin Lock Hospital; so that I think Mr. Guthrie must have been under some misapprehension, when he made the following observation: — "I honestly declare, I cannot recollect more than two cases any way resembling it having occurred to me, out of several thousand instances; I do not believe that the proportion of such cases is greater than one in five thousand."— Mr. G. in making this observation, must mean to confine himself to the commencement of the ulcer; and I do not marvel he has seen but two instances — I have seen but one; and I scarcely expect to see another, as it excites so little sensation, not so say pain, at this stage, that it may even escape the observation of the patient himself.

It seems Mr. Guthrie and I are agreed with respect to the treatment of the phagedenic ulcer, and of the formidable train of secondary symptoms I have stated to arise from it; and he even refers to cases (See p. 329 and 330 of his Remarks) in which he witnessed the primary phagedenic ulcer and its secondary symptoms, as I have described them, both existing together in the same patients. But he conceives I am wrong in attributing these secondary symptoms exclusively to the phagedenic primary ulcer. His words are, "I can bring dozens of well substantiated cases, in which the ulcers were at first without any sloughing and phagedenic characters for a considerable time, yet subsequently assumed them, *and were followed by the se-*

*condary symptoms which Mr. C. considers as the true constitutional ones of specific venereal phagedena."*

It would be presumption in me to maintain, at so early a stage of the present investigation, that the constitutional symptoms I have detailed as appertaining to the phagedenic ulcer, can never arise from any other form of primary ulcer; all that I have stated is the fruit of my experience, and that enables me to affirm, that in every instance in which I had an opportunity of tracing the constitutional symptoms in question to their primary ulcer, *that* ulcer always exhibited the phagedenic character—Mr. Guthrie brings forward his own experience in support of the contrary opinion; but acknowledges that the ulcers he refers to afterwards became phagedenic. I give him full credit for his assertions, and am certain the investigation of this intricate subject cannot be in better hands. But it is probable there is less difference of opinion on this head between us than he is inclined to imagine. He will, at least, do me the justice to allow that my cases are detailed with candour, since he has found in them wherewithal to assail the very opinions I grounded upon them; for so far from arranging them in support of a preconceived theory, I made no attempts at generalization until the cases were completed, and the circumstances they contained were carefully examined and compared with each other. But so solicitous was I in noting those cases, that if I entertained any doubts with respect to the nature of an ulcer, I endeavoured to convey my own uncertainty in the language in which I described it. Thus, in my last Essay, if Mr. Guthrie takes the trouble of looking at cases 28, 29, and 32, he will find the primary ulcers, which were followed by the phagedenic constitutional symptoms, cautiously described in the following words:—The first is "a small ulcer of foul appearance and phagedenic *character*, on one of the labia." The second, "a small foul ulcer *inclining* to phagedena.—The third an ulcer about the size of sixpence, exhibiting a white *sloughy* appearance, was situated on the body of the penis; the integuments of which were *undermined* to some little extent."

In all the other cases, where no doubt could exist with respect to the nature of the ulcer, it was, without circumlocution, termed a phagedenic ulcer. The doubtful ulcers were, I well recollect, covered with a white tenacious adherent matter; and after a time, *evinced a disposition to burrow*, with a phagedenic border, which, together with the constitutional symptoms that attend them, induced me to class them with the phagedenic. Perhaps, indeed, I was guilty of some remissness in not pointing my reader's attention to the language I employed in those different descriptions.

Under these circumstances, it is not improbable but that the cases which came under Mr. Guthrie's observation, were of this nature; and it would be highly advantageous, if he, as well as all future enquirers, would attend particularly to the characters of such primary ulcers as are followed by the constitutional symptoms, which I have, without circumlocution, termed phagedenic. It has been denied, that these constitutional symptoms exclusively originate from a phagedenic ulcer, but whatever may be the primary ulcer from which they arise, they indicate *to the practitioner* the most obstinate and dangerous of venereal diseases—one in which full courses of mercury are most decidedly injurious; but which is often benefited by the alterative plan: but even this I would postpone (except the safety of some important part was threatened) until the disease was evidently *on the wane*—a circumstance which is clearly indicated by any new cutaneous spots which occur, spreading into scaly blotches, instead of degenerating into foul malignant ulcers. And I may here be pardoned for repeating the valuable practical fact, that when an eruption is *scaly*, no matter whether it is so from the *commencement*, as in the syphilitic lepra and psoriasis, or in the *decline* of the disease, as in the papular, pustular, and tubercular eruptions, mercury may then be exhibited with *safety* and *effect*.

A few other objections still remain to be answered. Mr. G. observes, "In the first work we find, that the tubercular eruption is considered as a distinct constitutional symptom, and arranged as the fourth in order, and he was then disposed to attribute it to the burrowing ulcer. In the second work, we find he distinctly classes the pustular and tubercular eruptions under one head, and refers them to the phagedenic and sloughing ulcer."—My answer to this is very simple, and may be very brief. My experience was not stationary, and opportunities occurred of seeing pustules and tubercles in the very same patients, who were affected with primary phagedenic ulcers; add to this, that I had sufficient reason to consider the burrowing and phagedenic ulcers as the same species of complaint, because these ulcerations are very similar, and they produce the same constitutional symptoms. From another passage in the same page, I collect, that Mr. G. conceives

I am guilty of the inconsistency of overturning my own doctrine by the detail of cases of the spontaneous origin of tubercles in deranged constitutions, where there was no reason to suspect the communication of any morbid poison.

That tubercles, which terminate in ulcers, are produced by morbid poisons, and that they also occur simply in deranged constitutions, where no morbid poison has been imbibed, cannot, at present, be denied; though to account for the anomaly will not be so easy a task. But I do not see how I have overturned my own opinions in detailing these facts—as well might it be argued, that buboes do not arise from a venereal poison, because they sometimes originate in other causes.

To his arguments in support of the same objection, derived from an eruption both papular and pustular, existing at the same time, (p. 337 of Mr. G's Remarks,) I answer, that the eruption of case 59, as delineated in plate III. consists of ulcers covered with thick crusts and pustules, intermingled with papulæ. The character of the eruption was, however, obviously pustular, though papulæ were present; precisely in the same manner as the eruption in small-pox is pustular, though papulæ may be intermingled with the full formed pustules.

The case of sloughing ulcer, with constitutional ulcers, which I stated in my first publication as healing fnom their margins, is also urged against me in the same page, in corroboration of the same objection. It is true, I have pointed out as one of the distinctive marks of the constitutional ulcers of the phagedenic disease, their healing from their centre, and not from their margins. A peculiar character which I had so often observed in the secondary ulcers that arise from a primary phagedena, that I could not but regard it as a mark of great practical importance, which designated an obstinate and dangerous disease; but I did not, and do not affirm, in the present early stage of the inquiry, that this mode of healing is exclusively attendant upon the constitutional ulcers of a primary phagedena. My meaning is sufficiently evinced, by the following passage in my Synopsis: —"Eruption of tubercles, or spots of a pustular tendency, or both intermixed, preceded by fever, and terminating in ulcers covered with thick crusts, which *often* assume a conical form, healing from their centre, and extending with a phagedenic margin."—But the primary ulcer, in the above instance, might have been originally of my 2d Class—*that*

with high edges, which approaches to the phagedenic character, and have become sloughing through irritation; and if so, the constitutional ulcers would naturally heal from their margins. The enquiry is in its infancy, and should meet with that candid indulgence which its delicacy requires, while the strictest scrutiny need not be neglected.

"The difference of opinion, (Mr. Guthrie in concluding his remarks observes,) which exists between us, does not, I am happy to say, extend to the mode of curing these complaints, except in one instance. In the antiphlogistic treatment of the phagedenic, and common sloughing ulcer, I fully coincide; it is the practice which Dr. Ferguson recommended before Mr. Carmichael wrote, and which I have for some years pursued. I concur with him in the necessity of abstaining from the use of mercury in the earlier stage of the constitutional symptoms, whilst I bear the strongest testimony to its great utility in the latter, when administered with caution."

It is as gratifying to me as to Mr. Guthrie, that in the more material points—those of practice—we are agreed; although he is disposed to give Dr. Ferguson all the credit of this concurrence; still, however unwillingly, I must, in duty to myself, make some references to dates, in order to prove that I have not endeavoured to appropriate to myself the discoveries of any man.

The 4th Vol. of the Medico-Chirurgical Transactions, in which Dr. Ferguson's most interesting paper is inserted, is dated November 24, 1813, and it came to my hands early in 1814. The first part of my work was published in the beginning of January 1814; but cases are detailed of the successful employment of blood-letting, and the antiphlogistic means in painful ulcers attended with inflammation, which occurred so far back as 1811, 1812, and 1813. (See Cases 2, 3, 4, and 5, all of which were Hospital Cases under the eye of many of the profession.)

The Second Part of the same Work begins with my 4th Chapter, and was published in the latter end of 1814. In which it appears that the same treatment, that I had found so useful in inflammatory ulcers, I had also adopted in phagedenic ulcers, which are so generally painful and irritable; and many of these cases so treated occurred in the Lock Hospital, so far back as 1811, 1812, and 1813. See particularly Cases 48, 51, and 53. Many other cases to the same effect, I detailed in my public Lectures on the subject delivered at the Lock Hospital, in the winters of 1812 and 1813.

The object of Dr. Ferguson's candid and excellent paper is to inform us of the practice among the Portuguese surgeons, and of the successful issue of their mode of treatment, in which mercury is so little employed, and not to detail any improvements emanating from himself; and it was with no little satisfaction that, in the second part of my work, I was enabled to resort to his important testimony in support of my own views, at that time standing so much in need of such corroboration.

Mr Guthrie, I find, differs from me in one point of practice—the use of the lancet in the sloughing ulcer. This difference of opinion is, however, grounded not on his experience, but, as appears from the passage, p. 339, (where he compares the sloughing ulcer to the gangrenous toes of old people) on theoretical views. The practice I have recommended is supported by a sufficient experience of its utility; for these ulcers spread rapidly under the influence of wine and bark, yet cease their ravages on the occurrence of a spontaneous hæmorrhage from the part, or from the use of the lancet, and the antiphlogistic plan combined with the exhibition of opium or cicuta.

But, with respect to the existence of a plurality of poisons, each of which produces primary and secondary symptoms peculiar to itself, there are decisive facts to vindicate my adoption of this much canvassed opinion, and they are these—that in each of four classes, into which I have divided venereal diseases, I have witnessed the eruption, or other secondary symptoms, immediately succeeding the corresponding ulcer, or other primary symptoms, or both existing in the same patient at one and the the same time; and in no instance do I attempt to establish a class, in which it is not supported on those solid foundations. It is true, in one of those classes (the Pustular Venereal Disease) I have not had the good fortune to witness this conjunction more than half a dozen times. But in all the other classes, the evidence which in this way confirms them, is amply sufficient, and I will add in two of them superabundant. I trust, therefore, that I have perfectly satisfied Mr. Guthrie, that his *objections* to my theory, if theory it can be called, which depends no longer on speculation, but indisputable facts, are *not* sufficient to subvert it; but even if these facts were annihilated, it would be to me, as it must be to others, a matter of comparative indifference, as long as the practice I have recommended shall be considered as of value. Yet, as a step to that practice, I must acknowledge my obligations to the views I connected with them, and which led me at once to the beneficial measure of rejecting the prevailing prejudice of the medical world, and withholding the use of mercury in the generality of venereal disorders. This mode of treatment, chiefly in consequence of its successful adoption in the army, already prevails to an extent that could scarcely, in so short a period, have been hoped for; and (to give it no other name) the theory which was of such advantage to my labours, may possibly be no longer of any considerable importance, unless, indeed, it shall hereafter be discovered, that each peculiar species of venereal complaint will admit of a speedier cure by means of a remedy, that is less efficacious in those of a different character. But this is now of little moment, since every species of venereal disease can be combated by specified means, and with the fairest hopes of decisive success. Let me not, however, be understood as admitting, that the classification I have introduced (if that classification is miscalled my theory, for theory I have none) having accomplished its purposes, may now be abandoned. Its utility must hereafter be as great as heretofore, in developing and explaining the circumstances of anomalous cases; and may one day be a means of distinguishing those venereal diseases, with which we are at present acquainted, from those that may be imported among us from abroad, or which may possibly originate from very different causes, not necessary here to investigate or conjecture.

Without pluming myself, however, upon these considerations, I rest all my credit upon more substantial grounds, the practice to which they gave birth; and particularly the distinctive characters I have established for the more ready discrimination of malignant and destructive disorders from the milder and safer; the appropriate remedies I have experienced as useful, and some of which I was the first to adopt, particularly those extensive depletions which are now found of so much benefit under circumstances of inflammation; and, lastly, the simple and satisfactory criterion I have introduced into notice, by which it is ascertained when mercury may be employed with effect, and when it ought to be discarded as useless or pernicious.

I am, &c.

RICHARD CARMICHAEL.

---

POSTSCRIPT.

In the same Number of your Journal there is a Review of my Work; and although it is by no means my intention to cope, on all occasions, with my anonymous antagonists, yet in the present instance, as so favourable an opportunity offers while I have the pen in my hand, I shall venture briefly to reply to the few objections which are urged against me in this sharp but occasionally flattering critique. The Writer observes, p. 419, that I am "unnecessarily severe on those who have borrowed the term *Syphiloid* to express diseases which resembled Syphilis; but this severity is completely turned against himself in the Appendix, where he is forced to relinquish his opinion that true Syphilis differs from other venereal complaints by always requiring mercury for its cure." To this objection I have only to answer, that it never entered into my imagination to nominate or class diseases by the remedies required for their cure. I, perhaps unwisely, conceived that the more certain grounds for distinction or classification were the characters, phænomena, symptoms, and history of the diseases in question

In the same page, an error in expression is urged against me in the following passage, which arose from pure inadvertence. "The chancre remained a month stationary, neither increasing nor diminishing; but *at* the expiration of this time, a deep excavated ulcer *had* formed on the induration." Let the participle "had" be blotted out, and the sense of the passage is clearly what I intended to express, that after the expiration of a month, the chancre began to deepen in the induration. I acknowledge that without this explanation the sense is obscure, but the error must have occurred in a heedless and unsuccessful attempt to improve the language after the passage had been written.

The Reviewer seems to doubt that the two cases I have detailed of Chancre were truly Syphilitic. In reply I beg to state, that my opinion of the nature of the disease was grounded on the *induration* in the primary, and *scaliness from the commencement* in the secondary symptoms; that the cases are candidly and impartially detailed, in direct opposition to my own preconceptions; and that however opinions may vary with respect to their consequences, as MEDICAL FACTS they continue unshaken. I therefore do not conceive that I have been precipitate in "relinquishing my opinion, that true Syphilis differs from other venereal complaints in *always* requiring mercury for its cure." These two cases, if allowed to have been Syphilitic, are surely in themselves sufficient testimony that I have not been precipitate, although I by no means would be understood to advocate the treatment of true Syphilis without mercury, on the testimony of two or even two hundred cases. All I infer from these circumstances is, that even true Syphilis itself *may* be cured without the agency of mercury; but they at the same time evince, by the tediousness of the recovery, that it would in all probability have taken place in a much shorter period through the use of that medicine.

These cases occurred while the work was at press, and were given in an Appendix, in which I stated that they were as yet incomplete, but that I would communicate the result to the Profession, if any change or relapse should occur. For this there cannot be a better opportunity than the present. The subject of the first case, James Murray, was discharged the hospital on the 20th of July, 1818, apparently well. He however was readmitted on the 4th of August, in consequence of a reappearance of the eruption (Psoriasis Syphilitica). He was ordered the decoction and powder of sarsaparilla, and was discharged free from all appearance of complaints on the 28th of September; but returned again to the hospital on the 10th of November with some fresh spots on his back and arms. He was desired to shew himself again if they should increase, but he has not since returned.

The subject of the second case, Edward O'Brien, was discharged well on the 13th of August, since which time I have not seen or heard of him. I have not a doubt, however, that the disease in both would have been cured in a far shorter time had mercury been employed. The only alteration these cases have made in my opinions, I have stated in the Appendix alluded to: " that in thus relinquishing my opinion, that true Syphilis differs from other venereal complaints by always requiring mercury for its cure, it is necessary to reduce the doctrine I hold to this proposition: that, with respect to the use of that medicine, it differs from them only in not being injured but decidedly benefited by it, in *all* its symptoms and stages."* The Reviewer thinks these sudden changes indicate one of two things. " precipitation in publishing an opinion, or vacillation afterwards." There are others who would rather consider that they indicate the honourable sacrifice of opinion to truth.

A similar imputation is also applied to my Observations

---

* See Essay on the Uses and Abuses of Mercury, p. 219.

on Iritis, and with similar justice. When I proceeded on my Inquiry concerning Venereal Diseases, so far back as my appointment to the Lock Hospital of Dublin in 1810, I determined on treating all diseases which did not assume the characters of Syphilis, as laid down by John Hunter and Willan, without the exhibition of mercury. Among the number of syphilitic symptoms I acceded to the generally received opinion that Iritis was one; an opinion I the more readily embraced, from the rapid amendment which always attends it when mercury is used. Since my first publication, however, a great number of cases occurred, in which Iritis was accompanied by the papular eruption; and not one presented itself, in which the scaly Lepra or Psoriasis was present, and therefore I flatter myself that in stating the result of my observations and increasing experience, few will agree with the Reviewer, that precipitation or vacillation can fairly be laid to my charge.

It is also hinted, that my work has been pushed to an unnecessary extent by the multiplication of cases. I can assure my Reviewer, that if this be the fact, it did not arise from any devotion to "*the prevailing taste of the day,*" but because I really was ignorant of any other mode of establishing disputed facts than by a sufficient accumulation of satisfactory evidence.

